# Europium as an inhibitor of Amyloid-β(1-42) induced membrane permeation

**DOI:** 10.1016/j.febslet.2015.09.027

**Published:** 2015-10-24

**Authors:** Thomas L. Williams, Brigita Urbanc, Karen E. Marshall, Devkee M. Vadukul, A. Toby A. Jenkins, Louise C. Serpell

**Affiliations:** a School of Life Sciences, University of Sussex, Falmer, East Sussex BN1 9QG, UK; b Physics Department, Drexel University, Philadelphia, PA 19104, USA; c Department of Chemistry, University of Bath, Bath BA2 7AY, UK; d School of Medicine, Case Western Reserve University, Cleveland, OH 44106, USA

**Keywords:** Amyloid-β Peptide, Europium, GM1 ganglioside, Permeation inhibition, Alzheimer’s disease, Protein misfolding

## Abstract

Soluble Amyloid-beta (Aβ) oligomers are a source of cytotoxicity in Alzheimer’s disease (AD). The toxicity of Aβ oligomers may arise from their ability to interact with and disrupt cellular membranes mediated by GM1 ganglioside receptors within these membranes. Therefore, inhibition of Aβ–membrane interactions could provide a means of preventing the toxicity associated with Aβ. Here, using Surface Plasmon field-enhanced Fluorescence Spectroscopy, we determine that the lanthanide, Europium III chloride (Eu^3+^), strongly binds to GM1 ganglioside-containing membranes and prevents the interaction with Aβ42 leading to a loss of the peptides ability to cause membrane permeation. Here we discuss the molecular mechanism by which Eu^3+^ inhibits Aβ42-membrane interactions and this may lead to protection of membrane integrity against Aβ42 induced toxicity.

## Highlights

• Europium ions complex with GM1 gangliosides in phospholipid membranes.
• Europium ions cause inhibition Aβ–membrane interactions.
• Europium blocks an Aβ receptor protecting against membrane permeation.
• Discrete Aβ binding events correlate to specific membrane permeation events.

## Graphical abstract

**Figure:**
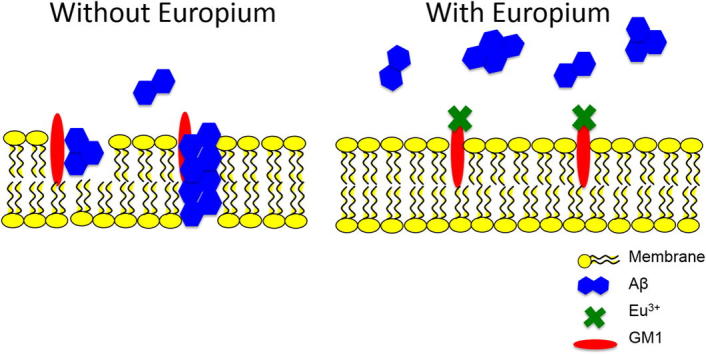

## 1 Introduction

Alzheimer’s disease (AD) is the most common form of dementia worldwide, with a global cost of over $600 billion in 2012 [1]. The pathological hallmarks of AD include the loss of neurons, the accumulation of neuritic plaques composed of extracellular, fibrillar Amyloid-beta peptide (Aβ), and the deposition of intraneuronal neurofibrillary tangles composed of tau [2]. Aβ_1–42_ (Aβ42) is the predominant variant deposited in AD brains.

The proteolytic cleavage of the integral membrane protein, Amyloid Precursor Protein (APP), at the N- and C-termini by β- and γ-secretase respectively, results in the release of Aβ [3]. This release of Aβ from its transmembrane location means that the peptide retains an ability to interact with and/or insert into biological membranes. The propensity for peptides to form amyloid fibers is promoted by the β-strand conformation forming stable networks of hydrogen bonds that result in characteristic cross-β structures [4]. The hydropathic properties and charges on specific amino acids in the polypeptide chain also influence the peptide’s ability to interact with surfaces such as membranes [5], [6], [7]. Therefore, subtle alterations in surface and membrane properties could dramatically affect both Aβ–Aβ and Aβ–surface interactions.

Biological membranes are composed of complex mixtures of phospholipids, proteins, and membrane receptors. Gangliosides are a type of glycosphingolipid found in the outer leaflet of eukaryotic membranes that are composed of a hydrophobic, membrane-embedded ceramide and a hydrophilic sialic acid moiety [8]. Gangliosides serve a variety of functions such as cell type-specific markers, differentiation and developmental markers, receptors, and as mediators of cell adhesion [9]. Expression of the GM1 ganglioside in the eukaryotic membrane is typically around 2% (*w*/*w*) [10], but can vary between 0.5% and 13% (*w*/*w*) depending on cell type and cell cycle stage [11], [12], [13], [14]. The GM1 within lipid membranes has previously been shown to significantly affect the affinity of Aβ42 for the membrane. We have previously shown that Aβ oligomers possess high affinity and high avidity to GM1-containing membranes [15]. In our previous study, we showed that Aβ oligomers cause increased permeation of the membranes, whereby the peptide causes the formation of well-defined holes and defects [15]. GM1 has been shown to significantly affect the assembly state and oligomerization of Aβ, and it was recently reported that increasing concentrations of GM1 induces a greater proportion of β-sheet structure within Aβ [16], [17]. GM1-bound Aβ was proposed to act as a seed in the fibrillization of the peptide, and fibril extension occurred via consecutive binding of soluble Aβ at the ends of the growing fibrils [18]. Therefore, GM1 may provide an ideal surface for Aβ adsorption via effective hydrophobic and electrostatic interactions, and blocking of this receptor could significantly affect both Aβ–membrane interactions and Aβ-induced permeation.

Europium and other lanthanide complexes have been used to detect neutral sugars and glycolipids in ovarian cancer diagnostics [19] because the metal center of the europium cooperatively co-ordinates to the oligosaccharide and sialic acid moiety of gangliosides [19]. Europium complexes have received much attention as both therapeutic and diagnostic agents, including as angiogenic inhibitors [20], in vivo neuroimaging agents [21], [22], as therapeutic agents in the treatment of ischemic heart disease [23], in myocardial infractions [24], and are reviewed in [25]. Williams et al. previously reported the potential use of europium (Eu^3+^) as a means of inhibiting the binding of cholera toxin to tethered large unilamellar vesicles (LUV) [26]. Here, we report the effect of Eu^3+^ complexed to GM1-containing membranes on the avidity and affinity of Aβ42 binding to membranes in vitro, and the ability of Eu^3+^-coordinated membranes to resist Aβ-induced membrane permeation. We also determine the sequence of events that occur during Aβ–membrane binding, permeation and GM1 mediated seeding during Aβ fibril formation.

## 2 Experimental procedures

### 2.1 Amyloid peptides

Aβ_1–42_ HFIP (referred to as Aβ42), >97% purity was purchased from rPeptide (Bogart, GA, USA). All peptides were used without further purification.

### 2.2 Peptide preparation

Aβ42 was prepared as described previously [27]. Briefly, 1 mg mL^−1^ Aβ42 was solubilized in 1,1,1,3,3,3-hexafluoro-2-propanol (HFIP) (Fluka, Sigma–Aldrich Company Ltd., Dorset, UK), vortexed for 60 s and bath sonicated for 60 s. Solvent was removed using a stream of dry nitrogen and vacuum desiccated for 30 min. Aβ42 was re-solubilized at 1 mg mL^−1^ with dimethyl sulfoxide (DMSO) >99.9% (Sigma–Aldrich Company Ltd., Dorset, UK), vortexed vigorously for 60 s and sonicated for 60 s. The peptide was buffer exchanged with 10 mM HEPES, 100 mM NaCl, 1 mM EDTA, and 0.05 mM NaN_3_ (all purchased from Sigma–Aldrich Company Ltd., Dorset, UK), and referred to as HEPES pH7.4, using a Zeba™ desalt spin column. The eluted peptide was centrifuged in a 4 °C microcentrifuge (Eppendorf UK Ltd., Cambridge, UK) at 16,000×*g* for 30 min and the concentration determined using a Biophotometer (Eppendorf UK Ltd., Cambridge, UK). This preparation has been previously characterized and confirmed to contain predominantly oligomeric Aβ42 [27], [28]. Freshly prepared Aβ42 was used for the experiments immediately, while Aβ42 fibers were prepared by statically incubating oligomeric Aβ42 at 21 °C for 24 h.

### 2.3 Europium III chloride and other lanthanide metal ion preparation

Europium III chloride (Eu^3+^), erbium III chloride, gadolinium III chloride, lanthanum III chloride, terbium III chloride and ytterbium III chloride (99.99% anhydrous, Sigma) were diluted to a stock concentration of 1 mM in HEPES pH 7.4, and then diluted to the working concentrations (10 and 100 μM).

### 2.4 Biomimetic membrane constituents

1,2-dimyristoyl-s*n*-glycero-3-phosphocholine (DMPC), 1,2-dimyristoyl-s*n*-glycero-3-phosphoethanolamine (DMPE), 1,2-dimyristoyl-s*n*-glycero-3-phospho-(1′-rac-glycerol) (DMPG), 1,2-dimyristoyl-s*n*-glycero-3-phospho-l-serine (DMPS) and 1-oleoyl-2-(12-biotinyl(aminododecanoyl))-s*n*-glycero-3-phosphoethanolamine (Biotin-PE) were purchased from Avanti Polar Lipid Inc. (Alabaster, AL, US). Cholesterol, 95% and Monosialoganglioside G_M1_ from bovine brain, >95% lyophilized powder were purchased from Sigma–Aldrich Company Ltd. (Dorset, UK). All materials were used without further purification. The phospholipid composition was previously used to study Aβ interactions with biomimetic membrane models [15]. This composition was used as it contains relevant phospholipid headgroups and sterols at realistic proportions to natural, biological membranes found in eukaryotic cells and ensures a relevant zwitterionic (phosphatidylcholine ∼50% and phosphatidylethanolamine ∼10%) and anionic (phosphatidylserine ∼2–5% and phosphatidylglycerol ∼2–5%) lipid ratio found within cellular membranes [29], [30], [31]. Cholesterol typically comprises around 30% of the eukaryotic phospholipid membrane [32], but can vary depending of the cell type [33], while GM1 is present at a molar ratio of around 2% [10].

### 2.5 Calcein-encapsulated large unilamellar lipid vesicles (LUVs)

Calcein encapsulated at a self-quenching concentration within the aqueous space of lipid vesicle lumen undergoes fluorescence dequenching as it diffuses into the external surrounding solution, resulting in an increase in calcein fluorescence intensity at 520 nm. Calcein-encapsulated LUVs were prepared as described previously [27]. Briefly, 40 mg mL^−1^ 68:30:2 (*w*/*w*) DMPC/cholesterol/GM1 lipid films were rehydrated to 10 mg mL^−1^ with 200 mM calcein in HEPES pH 7.4 (Sigma–Aldrich Company Ltd., Dorset, UK), and vortexed vigorously for 30 min. The resulting suspension was passed 19 times through an Avestin extruder fitted with two stacked 100 nm polycarbonate membranes (GC Technology Ltd., Bedford, UK). Non-encapsulated calcein was removed from the LUVs using a 1 mL sephadex G-50 minicolumn preparation and spun in a 4 °C controlled Mikro 22R centrifuge at 2000 RPM for 3 min to dispel the LUVs into the centrifuge tube. The eluted LUVs were washed a further two times. The LUVs were diluted to 1 mg mL^−1^ in HEPES pH 7.4, and stored at 4 °C until use. 100 μM Eu^3+^ was added to calcein loaded LUVs and mixed gently at 300 rpm for 30 min to allow complexation.

The encapsulation of calcein was confirmed in the fluorimeter by determining the release of the self-quenched calcein upon the addition of 0.5% (*v*/*v*) Triton X-100. If the calcein intensity did not exceed 100% of the starting fluorescence intensity prior to triton addition, then the LUVs were discarded. Percentage calcein release was calculated according to:

(1) $$\%\;\text{Release}\;=(F-F_{0})\times 100/(F_{\max}-F_{0})$$

where *F* is the measured fluorescence intensity of calcein at the specific time, *F*_0_ is the initial measured fluorescence intensity at time = 0 min, *F*_max_ is the maximum fluorescence intensity measured by the complete lysis of LUVs by the addition of the triton surfactant.

### 2.6 Fluorescence Spectroscopy

Fluorescence measurements were carried out on a Cary Eclipse fluorimeter (Varian Ltd., Oxford, UK). Samples were placed in a 1 cm path-length submicro quartz cuvette (Starna, Essex, UK) and the calcein fluorescence was monitored at various time points using an excitation wavelength of 490 nm. Calcein emission was monitored between 500 and 600 nm, with maximum fluorescence intensity at around 520 nm at a controlled temperature of 21 °C. Excitation and emission slits were both set to 10 nm, and a scan rate set to 100 nm/min with 0.833 nm data intervals and an averaging time of 0.55 s. The photomultiplier tube was set to 400 V. Experiments were carried out in duplicate to confirm trends. Fluorescence intensities at the peak of 520 nm were plotted against time.

### 2.7 SPFS tethered large unilamellar vesicles

40 mg mL^−1^ stock solutions of the bilayer constituents were solubilized in 2:1 (*v*/*v*) chloroform/methanol and stored at −20 °C until use. A mixed lipid solution was prepared in a glass vial from the stock solutions containing (50:13:2:2:30:2:1 *w*/*w*) DMPC/DMPE/DMPG/DMPS/Cholesterol/GM1/Biotin-PE and the solvent was removed using a stream of dry nitrogen and vacuum desiccated overnight. The lipid films were rehydrated with 5 μM BODIPY dye (6-(((4,4-difluoro-5-(2-pyrrolyl)-4-bora-3a,4a-diaza-sindacene-3-yl)styryloxy)acetyl) aminohexanoic acid, sulfotetrafluorophenyl ester, sodium salt) (excitation wavelength = 648 nm, emission wavelength = 660 nm) (Molecular Probes, Invitrogen Ltd., UK) hydrolyzed to form the carboxylic acid dissolved in HEPES pH 7.4, and vortexed vigorously for 30 min. BODIPY is not a self-quenching dye at the concentrations used in these experiments, therefore is applicable to use with the SPFS system. The resulting suspension was passed 19 times through an Avestin extruder fitted with two stacked 100 nm polycarbonate membranes (GC Technology Ltd., Bedford, UK). Changes in mass (ng mm^−2^) at the sensor surface are calculated using:

(2) $$(A)=\frac{\Delta \theta }{\left(\Delta \theta /\sigma \right)}$$

where (*A*) is the absorbance in ng mm^−2^, (Δ*θ*/*σ*) is the surface concentration correlation of 0.1868° and Δ*θ* is the shift in resonance minimum. The fluorescence flux through the permeated membranes (D^*^) were calculated as previously described [15], and the *K_D_* (Eq. (4)) was calculated using the *k_off_* (Eq. 3a) and *k_on_* (Eq. 3b) dissociation and association equations, respectively using Origin 7 data analysis software (OriginLab):

(3a) $$R_{t}=R_{0}\exp-k_{\mathrm{off}}t$$

(3b) $$\frac{{\mathrm{dR}}_{t}}{\mathrm{dt}}=k_{\mathrm{on}}{\left[P\right]}_{t}R_{\max}-R_{t}(k_{\mathrm{on}}{\left[P\right]}_{t}+k_{\mathrm{off}})$$

where

(4) $$K_{D}=k_{\mathrm{off}}/k_{\mathrm{on}}$$

The equilibrium dissociation constant (*K_D_*) was determined, and all changes in reflectivity were normalized. The apparent dye diffusion coefficient (D^*^), that is a product of the fluorophore flux through permeated membranes, is calculated from the linearized change in fluorescence over time (extrapolated linearized slope of the dye diffusion rate), and takes into account vesicle membrane internal volume and membrane area and thickness.

### 2.8 SPFS bilayer preparation

For SPFS, the procedure for tethering of the LUVs can be found in detail in Ref. [15]. Briefly, gold-coated SFL6 glass were immersed in an ethanolic solution of 0.05 mM 11-mercaptoundecanoic-(8-biotinoylamido-3,6-dioxaoctyl) amide and 0.95 mM 11-mercapto-1-undecanol (99%, Sigma) and allowed to self-assemble onto the gold substrates for 16 h. The biotin surface was coupled with streptavidin (500 nM in HEPES pH 7.4). The biotin tagged vesicles were capture to the streptavidin surface and binding followed by SPR. Vesicles were tethered for 30 min before rinsing in buffer to remove no encapsulated fluorophore and non-bound vesicles from the system. A detailed schematic of the SPFS setup, flow-cell setup and extensive background to SPFS principles can be found in [34]. A 1 mM stock of europium III chloride in HEPES pH 7.4 was prepared, and then diluted to the working concentration (10–100 μM) prior to injection into the flow cell and allowed to adsorb to the GM1-containing LUVs for 30 min prior to rinsing of the flow-cell with fresh HEPES pH 7.4. Solutions were circulated around the flow-cell at a rate of 1.8 mL min^−1^ using a peristaltic pump for optimized analyte delivery, while minimizing the mass transport effects and the shear force on the bilayer vesicles.

### 2.9 Transmission electron microscopy

A 4 μl droplet of the peptide (80 μM) was adsorbed onto formvar/carbon coated 400 mesh copper grids (Agar Scientific, Essex, UK) for 60 s, and blotted dry. 4 μl of 0.22 μm filtered water was added to the grid and immediately blotted, then negatively stained with 4 μl of 2% uranyl acetate adsorbed for 60 s and blotted dry. The grid was allowed to air dry before examination on a Hitachi 7100 microscope (Hitachi, Germany) fitted with a Gatan Ultrascan 1000 CCD camera (Gatan, Abingdon, UK). Aliquots of samples at the stock concentration were taken at time points for each experiment to monitor fibrillization. Measurements were made using ImageJ [35].

## 3 Results

### 3.1 Solubilized Aβ42 forms small, soluble oligomers that assemble to form fibers

Transmission electron microscopy (TEM) confirmed the oligomeric and fibrillar morphology of the Aβ42 peptide following dissolution and then after 24 h incubation, respectively. A substantial in depth characterization of both peptide preparation protocol and Aβ–membrane permeation has previously been carried out using calcein release, TEM, circular dichroism, SPFS, and atomic force microscopy [15], [27]. We showed that Aβ42 used immediately after preparation formed small, circular oligomeric assemblies ranging in size between 1 and 5 nm, [15], [27]. Electron micrographs of 24 h incubated peptide showed larger assemblies composed of curvilinear oligomers, and small protofibrils ranging between 15 and 30 nm, and small fibers ranging between 140 and 240 nm in length and 5 nm in width [27]. Moreover, Aβ oligomers in the presence of Eu^3+^ were observed to form fibrils indistinguishable from fibrils formed in the absence of Eu^3+^, therefore, Eu^3+^ does not adversely affect the morphology of the assembling peptides or alter the aggregation pathway (Supplementary Fig. 1).

### 3.2 Selection of Eu^3+^ as inhibitory agent as opposed to other lanthanide ions

**Fig. 1:**
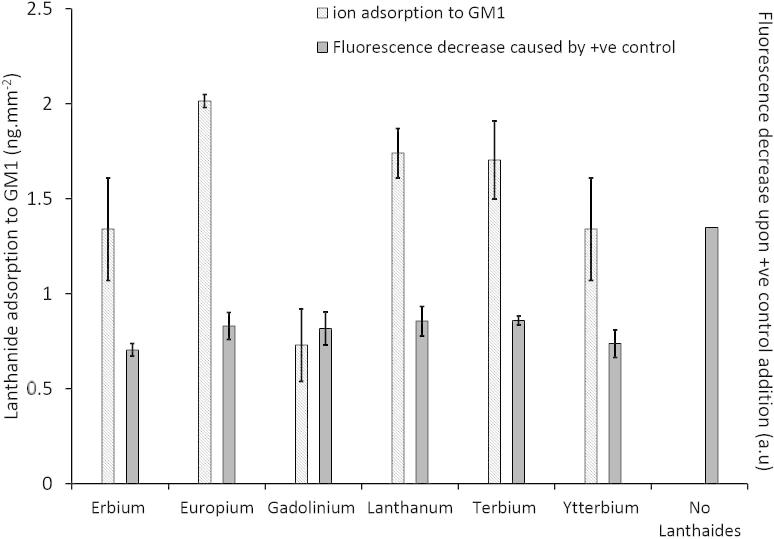
Mass adsorption of lanthanide ions to GM1-containing LUVs and total proportion of BODIPY release. Comparison of the mass of various lanthanide metal ions adsorbed to GM1-containing LUVs, showing a higher mass adsorption of europium trivalent metal ions compared to the other lanthanide ions tested (primary *y*-axis). Addition of 10 μM positive control cholera toxin (*Ctx*) show that all lanthanides tested cause ∼40% decrease in permeation compared to when lanthanides are not present (secondary *y*-axis).

Surface Plasmon field enhanced Fluorescence Spectroscopy (SPFS) was used to screen potential candidate lanthanide ions for complexation to GM1-containing membranes. Initially, increasing concentrations (0–10 μM) of the positive control cholera toxin (*Ctx*) were added to the tethered LUVs that had no lanthanide metal ions coordinated, and the mass adsorption of the control protein measured (Supplementary Fig. 2). This measurement was to determine the appropriate concentration of *Ctx* to add to the lanthanide coordinated LUVs for comparison. A concentration dependent increase in *Ctx* adsorption was observed and when equilibrium was reached at 10 μM *Ctx* equilibrium. Therefore, 10 μM *Ctx* was used to ensure a like-for-like comparison between the lanthanide species. Erbium III chloride, europium III chloride, gadolinium III chloride, lanthanum III chloride, terbium III chloride or ytterbium III chloride were added to the tethered membrane vesicles and allowed to complex with the membrane, and the amount of metal ion adsorption determined (Fig. 1). To test the abilities of the lanthanide ions to resist peptide-induced permeation, *Ctx* was injected into the system and the permeation determined (Fig. 1). The *Ctx* specifically associates with gangliosides, therefore this positive control was used to ensure that maximal permeation of the membranes was observed in order select the most appropriate lanthanide ion to use in this study.

The addition of the six different lanthanide metal ions resulted in varying levels of adsorption to the GM1 containing membranes (Fig. 1). Gadolinium showed the lowest affinity binding to the tethered membranes, followed by ytterbium and erbium. Terbium showed the third highest amount of metal ion binding to the membranes, followed by lanthanum. The highest amount of metal ion binding was observed with europium ions (Fig. 1). ANOVA statistical analysis shows a significant difference between the groups of the metal ions in the amount of metal ion binding to the GM1-containing membranes (*P *< 0.05).

The addition of the 10 μM *Ctx* to lanthanide-complexed membranes resulted in a significant reduction in permeation of the membranes compared to *Ctx* alone (Fig. 1). All lanthanides tested using the positive control *Ctx* toxin resulted in similar abilities to reduce membrane permeation. ANOVA statistical shows no significant difference between the lanthanides ability to cause reduced permeation (*P *> 0.05). However, there is a statistical significance between the lanthanides ability to reduce permeation compared to when no lanthanides are present (*P *< 0.05), whereby on average the lanthanide complexed LUVs showed a 40% reduction in permeation compared to when no lanthanides were present. We selected europium ions in this study because it showed the highest mass adsorption to the membranes, while similarly causing a reduction in the ability for the positive control to cause membrane permeation. Selection of europium trivalent ions appeared as the most suitable candidate to ensure the greatest binding to the biomimetic membranes for the subsequent studies using the Aβ peptide to permeate membranes. As each of the lanthanide ions had the ability to resist peptide-induced permeation, it would be intuitive to use the lanthanide with the greatest mass adsorption. It should be noted that Aβ42 may bind to varying degrees to the other lanthanide ions but as a proof of concept that lanthanide ions resist Aβ association to the membranes we dedicated this study to a single lanthanide ion to fully study the lanthanide-Aβ molecular mechanism.

The addition of 10 μM *Ctx* to 0–500 μM Eu^3+^ resulted in concentration dependent saturation of the membrane surface (Supplementary Fig. 3). Increasing the coordination of Eu^3+^ above 10 μM resulted in a very slight increase in *Ctx* adsorption to the membrane surface compared to membranes coordinated with 10 μM Eu^3+^. This could be an effect of oversaturation of the membrane surface that results from a kind of steric hindrance between the metal ions overcrowding the GM1 receptors.

### 3.3 Eu^3+^ complexed to LUVs in solution inhibits oligomeric Aβ42 induced permeation

**Fig. 2:**
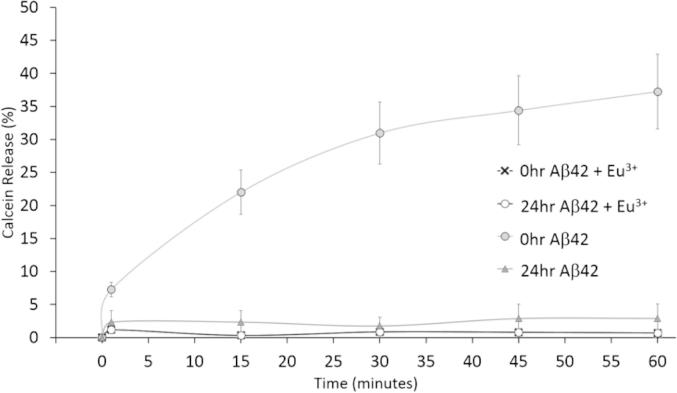
Calcein release assay monitoring the permeation of 0-h (oligomer) and 24-h (fibrillar) incubated Aβ42 permeation of LUVs. 10 μM 0 h- or 24 h-incubated Aβ42 was added to 100 μM Eu^3+^-coordinated LUVs and to LUVs alone. Calcein fluorescence was monitored for 60 min until equilibrium was reached. Addition of Aβ42 to Eu^3+^-complexed LUVs show decreased permeation of membranes in solution. Data was normalized and plotted as percentage released.

We have previously shown that lipid bilayers containing GM1 are highly susceptible to permeation by oligomeric Aβ42 [27]. A calcein release assay was employed to observe Aβ-induced permeation of LUVs in solution of a simple membrane composition. The addition of oligomeric Aβ42 alone resulted in an immediate release of 37% of the total encapsulated calcein from the LUVs (Table 1), indicative of Aβ-induced membrane permeation (Fig. 2) and in agreement with previous results [27]. Next, LUVs were preincubated with Eu^3+^ and this was followed by the addition of oligomeric Aβ42. Eu^3+^-complexed membranes resulted in the release of only 0.7% of the total encapsulated calcein (Fig. 2 and Table 1). Therefore, incubation of the Aβ42 with the Eu^3+^-coordinated LUVs showed a 56-fold decrease in membrane permeation relative to LUVs incubated in the absence of Eu^3+^ (Fig. 2).

Fibrillar Aβ42 has previously been shown to cause less permeation to membranes than oligomeric Aβ42 [27]. The addition of fibrillar Aβ led to 3% of the total encapsulated calcein release from LUVs in the absence of Eu^3+^ (Table 1), significantly less than permeation induced by oligomeric Aβ42. The addition of fibrillar Aβ42 to Eu^3+^-complexed LUVs resulted in the release of 0.7% of the total encapsulated calcein within the membranes, which corresponded to a 4-fold decrease in permeation. These results demonstrate that Eu^3+^ protects the membranes from permeation by both oligomeric and fibrillar Aβ42.

### 3.4 High affinity complexation between Eu^3+^ and GM1-containing membranes

**Fig. 3:**
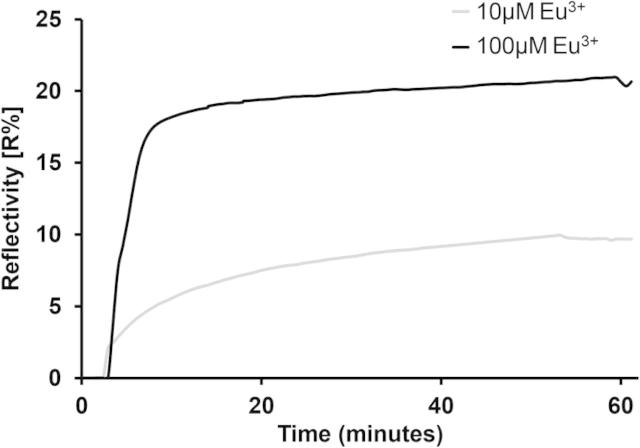
SPR measurement of 10 and 100 μM europium adsorption to GM1-containing LUVs. The addition of 10 and 100 μM europium III chloride to tethered GM1-containing LUVs resulted in a relatively high affinity interaction between the GM1 and the metal ion.

In order to investigate the membrane protective mechanism of Eu^3+^ against Aβ, Surface Plasmon resonance was used to follow binding of Aβ42 to tethered LUVs in the presence and absence of Eu^3+^. Initially, the affinity of the Eu^3+^ with GM1 containing membranes was considered. Two concentrations of Eu^3+^ (10 or 100 μM) were injected into the flow-cell, and incubated with the GM1-containing LUVs until equilibrium was reached. The adsorption of 10 and 100 μM Eu^3+^ resulted in a 0.39° and 1.46° up-shift in angle-resolved resonance minima, respectively. This change in the resonance minima equates to the adsorption of 2.09 ng mm^−2^ (±0.69) of 10 μM and 7.82 ng mm^−2^ (±0.59) of 100 μM Eu^3+^, calculated using the equation (*A*) = Δ*θ*/(Δ*θ*/*σ*). Adsorption of 10 μM Eu^3+^ to the tethered LUVs resulted in an equilibrium dissociation constant of *K_D_ *= 0.48 μM (±0.04) and the adsorption of 100 μM Eu^3+^ resulted in a *K_D_ *= 0.63 μM (±0.18) (Fig. 3), which represents strong binding of a similar strength to the specific binding between cholera toxin and GM1 [26].

### 3.5 Eu^3+^-complexed to LUVs inhibits the interaction of oligomeric Aβ42 to the membrane surface

Surface Plasmon field-enhanced Fluorescence Spectroscopy (SPFS) was used to concurrently measure Aβ adsorption to the membrane surface, the avidity of these interactions and the fluorescence flux through the permeated membranes [15], [26].

**Fig. 4:**
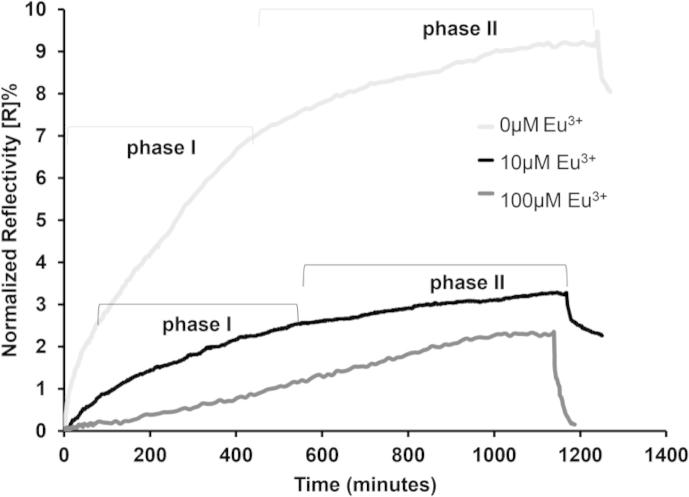
SPR measurement of oligomeric Aβ42 adsorption to 0, 10, and 100 μM europium-coordinated LUVs. The addition of oligomeric Aβ42 (10 μM) to Eu^3+^-complexed tethered LUVs and the binding of the peptide to the membrane surface was monitored. Increasing concentrations of Eu^3+^ resulted in significant decrease in Aβ42 binding. The exponential phases for each europium concentration associated with the determined biphasic exponential functions are included and reported in Table 3.

10 μM oligomeric Aβ42 was injected into the flow-cell and the change in mass monitored. 10 μM Eu^3+^ significantly reduced Aβ42 adsorption to the tethered LUVs, while 100 μM Eu^3+^ almost entirely prevented the adsorption of Aβ42 (Fig. 4). The Eu^3+^ and Aβ42 mass binding and equilibrium dissociation constants are shown in Table 2. The adsorption of 10 μM Eu^3+^ resulted in a 6% decrease in the binding between Aβ42 and the LUVs, whereas the addition of 100 μM Eu^3+^ resulted in a 94% decrease in binding between Aβ42 and the LUVs. The peptide was non-specifically adsorbed to the membrane surface and was easily washed off into solution. Thus, the addition of 100 μM Eu^3+^ to LUVs inhibited the ability of Aβ42 to bind to membranes in a concentration dependent manner.

The association binding data (*k_on_*) were further analyzed by fitting to mathematical exponential curve model to determine the number of analyte-ligand binding events that occur during the binding of Aβ to the LUV surface, and these values are summarized in Table 3. The exponential fitting function provides a means to statistically and mathematically determine the best fit for the binding between Aβ and the membranes, and provides a more sophisticated fitting procedure that can distinguish curves that do not follow the simple bimolecular binding. A monophasic response (single exponential curve function) indicates that a single 1:1 binding event between the analyte (Aβ) and ligand (LUV) is occurring, such as the binding of single Aβ molecule to one GM1 receptor. A biphasic response (2 exponential curves) indicates two distinct and different binding events occurring during the association of Aβ to the membrane surface, for example, the specific association of the Aβ peptide to the GM1 receptor followed by GM1 induced seeding fibrillization of Aβ on the membrane surface, as has previously been reported [18].

From the model fitting and the analysis of the Aβ42 oligomer-membrane binding in the absence of europium (0 μM Eu^3+^), it was revealed that there were two distinct binding events between Aβ42 and the membranes (show as t1 and t2 in Table 3), because the best curve fit was found to be to two consecutive exponential curves. Phase (I) the initial 1:1 specific binding between Aβ42 to the GM1-containing membranes (t1). This is then followed by Phase (II), non-specific binding of Aβ42 to the membranes and/or Aβ elongation as a result of GM1-induced seeded fibrillization (Fig. 4). Because the *binding* is relatively strong, a non-specific adsorption of Aβ to the LUVs is unlikely. Self-assembly and fibrillization of Aβ42 on the membrane surface would be more in line with the observed *binding*, and we hypothesize that the specific Aβ-bound GM1 is acting as a seed for further Aβ molecules to aggregate. The first event occurs between 11 and 430 min (Phase I, t1), and corresponds to the immediate binding of the Aβ to the GM1-containing membrane surface (Fig. 4 and Table 3). The second exponential curve fits from the beginning of 430 min (Phase II, t2), which corresponds to the GM1-Aβ complex acting as a seed for further Aβ fibrillization (Fig. 4 and Table 3).

The binding of oligomeric Aβ42 to 10 μM Eu^3+^ complexed-LUVs revealed that the best fit was also obtained using the biphasic exponential function, therefore, two binding events are occurring similar to when no europium is present. The first binding phase occurs between 60 and 505 min (t1 Table 3), corresponding to the initial binding of Aβ to the membrane surface (Fig. 4), but took significantly longer to occur compared to when europium was absent (81% increase in the initial t1 time phase) (Table 3). This is attributed to the low concentration of europium blocking a significant proportion of the GM1 binding sites and also altering the net charge of the membrane surface, therefore, the Aβ took longer to bind to the membranes because of the alteration in membrane surface elicited by the low concentration of europium. The second phase occurs at 505 min corresponded to the beginning of the Aβ fibrillization resulting from the GM1-seeding effect (Fig. 4 and Table 3).

Analysis of Aβ42 association to 100 μM europium coordinated membranes showed a poor fit to a mono-, bi- and tri-phasic exponential fitting and were therefore all excluded. This showed that Aβ42 did not bind significantly to 100 μM Eu^3+^ coordinated LUVs, suggesting that the Aβ binds non-specifically to membranes coordinated with high concentrations of Eu^3+^, as all the GM1 binding sites are occupied by the metal ions and there is a significant alternation in membrane charge caused by the positive metal ions that results in electrostatic repulsion between the Aβ and the membrane surface. The exponential model fitting calculations shows that increasing Eu^3+^ concentrations significantly affected the binding of Aβ42 to membrane surfaces, and that Eu^3+^ caused an increase in the time taken for Aβ42 to initially associate with LUVs. 10 μM Eu^3+^ caused a 5.4 fold delay in the initial association and formation of defects in the membrane compared to when Eu^3+^ was absent (Table 3, t1 and t2).

### 3.6 Eu^3+^-complexed to LUVs inhibits oligomeric Aβ42-induced permeation of LUVs

**Fig. 5:**
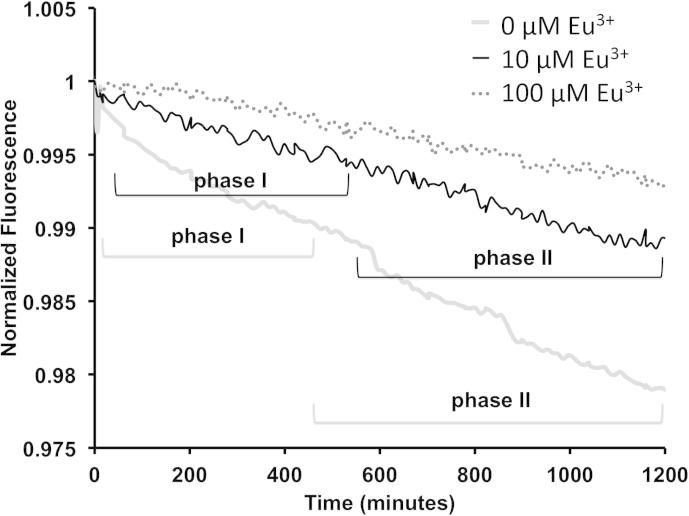
SPFS measurement of oligomeric Aβ42-induced permeation of 0, 10, and 100 μM europium-coordinated LUVs. Dye diffusion of the BODIPY induced by the addition of oligomeric Aβ42 (10 μM) to Eu^3+^-complexed tethered LUVs and the permeation of the membranes was monitored by SPFS as changes in fluorescence. Increasing concentrations of europium resulted in significant decrease in Aβ42-induced permeation. Included are the exponential phases for each europium concentration associated with the determined biphasic exponential functions reported in Table 2.

The addition of oligomeric Aβ42 to Eu^3+^ complexed LUVs resulted in decreased rates of BODIPY fluorophore diffusion (Fig. 5). A simple theoretical model was previously developed to fit the decrease in fluorescence−time curves (apparent dye diffusion coefficient (*D^∗^*)), and detailed in Ref. [26]. The addition of Aβ42 oligomers to LUVs resulted in *D*^*∗*^ = 1.72 × 10^−15^ m^2^ s^−1^ (±1.5 × 10^−15^). In contrast, when Aβ42 oligomers were added to 10 μM Eu^3+^-complexed LUVs, the *D*^∗^ fell to *D*^*∗*^ = 2.18 × 10^−16^ m^2^ s^−1^ (±1.2 × 10^−16^). Therefore, 10 μM Eu^3+^ coordinated LUVs resulted in an 87% decrease in membrane permeation rate. 100 μM Eu^3+^ complexed LUVs resulted in *D*^*∗*^ = 3.92 × 10^−17^ m^2^ s^−1^ (±2.69 × 10^−17^), showing an 82% decrease in permeation compared to in the absence of Eu^3+^ and a 98% decrease compared to 10 μM Eu^3+^ coordinated LUVs (Table 2).

Interestingly, although the 10 μM Eu^3+^ did not appear to have a strong effect on binding of Aβ42 to tethered LUVs, this lower Eu^3+^ concentration still had a strong effect on the peptide binding lag-phase and peptide assembly on the membrane (Table 3). There was a significant effect of Eu^3+^ on resulting BODIPY diffusion rates, which may indicate a link between self-assembly of Aβ42 on the membrane and the mechanism of Aβ42-induced membrane permeation. We posit that as Aβ42 begins to self-assemble into higher order oligomers (phase II Fig. 4, Fig. 5), diffusion of the fluorophore is reaching equilibrium and permeation is completed.

## 4 Discussion

The interactions between Aβ42 and membranes have been hypothesized to play an important role in the mechanism of cytotoxicity in AD. Our previous work showed that soluble oligomeric Aβ42 binds to GM1-containing membranes and causes the permeation of phospholipid membranes [15], [27]. Here, we demonstrate that (i) Eu^3+^ shows the greatest mass adsorption to GM1 containing membranes compared to the other lanthanide ions tested, and all the ions tested share similar abilities to resist *Ctx*-induced permeation in control studies; (ii) the interaction between Aβ42 and membranes is inhibited by the formation of complexes formed between Eu^3+^ and GM1 gangliosides; (iii) Calcein-encapsulated LUVs incubated with Eu^3+^ prevented the permeation of the membranes caused by Aβ42; (iv) Aβ42 showed decreased binding to Eu^3+^ preincubated membranes, consequently inhibiting Aβ42 membrane-induced permeation; We are also able to correlate the events that coincide between Aβ binding, permeation of the membranes and disturbance of biomimetic membrane homeostasis. This is highly significant as these events have not been previously measured simultaneously and directly linked, giving us a clearer picture of the molecular events associated with the binding and permeation of membranes by Aβ.

**Fig. 6:**
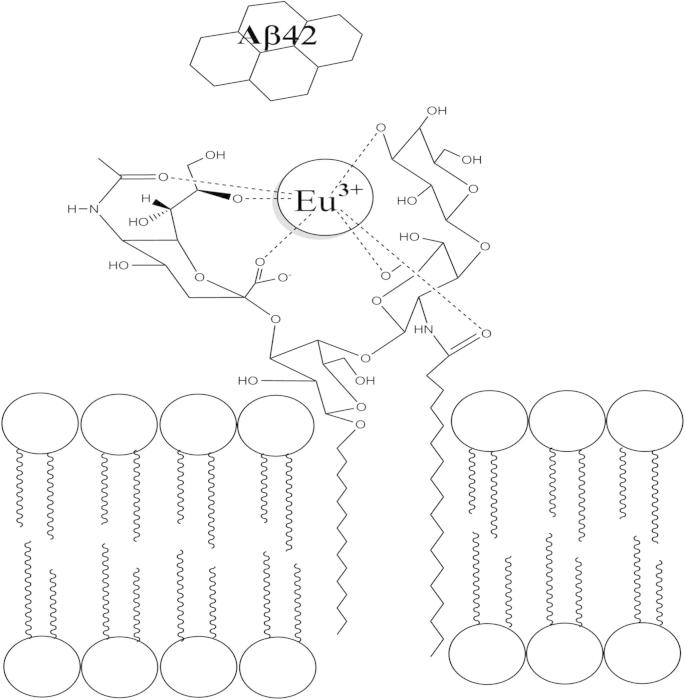
Schematic diagram of the proposed mechanism of europium inhibition of Aβ binding to membranes. The administration of europium blocks Aβ42 from interacting with the GM1-containing membranes, and inhibition of subsequent membrane permeation. Europium is proposed to selectively form cooperative complexes with the oligosaccharide and sialic acid residues via the europium metal center via electrostatic and hydrogen bonds that results in a coordination shell. The schematic is adapted from [19] and is not to scale.

The calcein release assay and BODIPY diffusion from tethered LUVs reveals that Eu^3+^-complexed LUVs resist Aβ42-induced permeation by reducing the binding of Aβ42. Our results highlight the significant role for GM1 and membrane properties in the mechanisms of Aβ toxicity. Arising from the findings reported here, a schematic of a proposed mechanism of Eu^3+^ inhibition of Aβ42-membrane interactions is shown in Fig. 6. GM1 has been shown to play a pivotal role in Aβ42-membrane interactions and Aβ42-induced permeation. The polar moiety of the GM1 provides a complementary surface for the polar amino acids to allow the formation of hydrogen bonds. GM1 gangliosides also provide a site of Aβ attachment to membranes [36], [37], [38], [39], and Aβ has been shown to colocalize in GM1-rich lipid raft microdomains [39]. Eu^3+^ was previously shown to covalently interact with the oligosaccharide and sialic acid moiety of the GM1 [19]. Therefore, if Eu^3+^ is blocking the GM1 site, it is no longer available to participate in Aβ interactions, aid Aβ polymerization that may lead to permeation of membranes. These results underline the importance of the Aβ toxic mechanism being mediated by GM1.

The decrease in permeation upon the addition of Aβ42 to Eu^3+^-coordinated membranes may be attributed to the change in the membrane charge resulting from the coordination of the positive trivalent Eu^3+^ ions to the membrane surface. Increasing the attractive electrostatic interactions between Aβ and GM1 results in increased insertion pressure due to the Aβ penetration into the membrane surface [40]. The solvent exposed aromatic residues of Aβ are able to stack onto the sugar rings of the GM1 driven by the net positive charge of the GM1 sugar rings that are in close proximity to the π-electron cloud of the amino acid aromatic ring [41]. Here, the positively charged Eu^3+^ ions would cause electrostatic repulsion toward the highly solvent exposed arginine residue 5 of Aβ42, which lies next to the aromatic phenylalanine at position 4 that can form stacking interactions with the GM1 sugar rings. By changing the net charge of the membrane with the coordination of positively charged Eu^3+^, the N-terminus of Aβ42 is repelled from the membrane surface. It has been demonstrated that dipolar compounds that shield the membranes negative charge can prevent the association of Aβ peptides with membranes and decrease Aβ-induced cell toxicity [42], similar to the mechanism we propose here to occur with Eu^3+^.

**Fig. 7:**
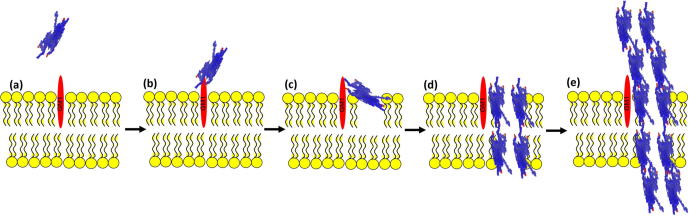
Schematic of the phases that occur during Aβ42-induced membrane permeation. (a and b) Aβ42 in close proximity to the GM1-containing membrane surface becomes associated with the GM1 gangliosides with high affinity (as determined by SPR Fig. 4). (c) Aβ42 begins to permeate the first lipid bilayer within 30 s of contact with the membrane (as determined by AFM in Ref. [15]). (d) Aβ42 permeated through both leaflets of the membrane bilayer and allows non-specific diffusion between the internal space into the extracellular environment (as demonstrated by SPFS Fig. 5). (e) Aβ assembly and fibrillization occurs, whereby Aβ42-bound GM1 acts as a seed for further Aβ42 assembly and the formation of higher order oligomers and fibers. (a–d) Represents the specific interactions of Aβ to the membrane surface and initial permeation events as demonstrated in Phase I (Fig. 4, Fig. 5). (e) Represents the initiation of Aβ42 assembly/fibrillization and fluorophore diffusion equilibrium events as demonstrated in Phase II (Fig. 4, Fig. 5). The addition of Eu^3+^ results in the blocking of the GM1 gangliosides, therefore inhibiting both Phases I and II by blocking the specific Aβ–membrane interactions and altering the membrane surface properties.

The mechanism by which Aβ causes membrane damage has been proposed to occur via three potential mechanisms; pore-formation, detergent-like and carpeting [43]. SPFS measures changes in mass in real-time, therefore, any subtle changes are monitored and visualized simultaneously. If the addition of oligomer Aβ42 resulted in total or partial lysis or removal of the lipid bilayer by a detergent-like mechanism, this would be observed as a decrease in the SPR signal. However, this is not observed (Fig. 4), therefore, a detergent-like mechanism of membrane permeation is not supported by our data. We have previously shown that Aβ42 rapidly forms defects and holes in planar phospholipid bilayers [15]. In support of this, here we demonstrate that the Phase I of Aβ42 binding to the membranes continues for 400 min. This establishes that, even though the initial formation of defects occurs very quickly, Aβ continues to bind to the membranes for a much longer time causing the formation of stable defects within the membrane. Phase II of binding and fluorophore diffusion that we attribute to Aβ self-assembly and fibrillization, would therefore not heavily influence the formation of membrane defects and permeation processes because they have already occurred much earlier during Phase I. This may suggest that mechanistically, the initial binding of Aβ and the primary formation of membrane defects are the critical determinant in Aβ-induced permeation of the membranes, and that Aβ self-assembly and fibrillization that occur during phase II are of less importance. We hypothesize that the initial binding of Aβ and formation of defects that correspond to Phase I (Fig. 4) is the time period where the most significant damaging events occur. These initial defects during Phase I are significant enough to cause dramatic membrane damage. We attribute Phase II (Fig. 4) to Aβ self-assembly and fibrillization, and during this time period, membrane homeostasis has already been significantly disrupted. A schematic of these events is shown in Fig. 7. Therefore, prevention of the interaction of Aβ with membranes could be an important factor in preventing and reducing Aβ membrane damage.

Here we show that lanthanide ions, and in particular Eu^3+^ represents a potential Aβ–membrane interaction inhibitor that could provide a novel strategy of altering the course of Aβ42 assembly and aggregation on the membranes and may effectively reduce the cytotoxicity associated with Aβ42.

## Figures and Tables

**Table 1:** Percentage of total calcein released from encapsulated membranes showing the effect of europium complexation on Aβ42-induced membrane permeation. Comparison of the membrane permeation effects of Aβ42 as a result of Eu^3+^ complexation to the GM1-containing membranes. Eu^3+^ causes a significant inhibitory effect toward both oligomeric and fibrillar Aβ42-induced membrane permeation (± are the standard error of the mean.

LUV administered samples	% Total calcein release
0 h (oligomers) Aβ42 + Eu^3+^	0.67% ± 0.16
24 h (fibrils) Aβ42 + Eu^3+^	0.70% ± 0.18
0 h (oligomers) Aβ42	37.23% ± 4.26
24 h (fibrils) Aβ42	3.00% ± 2.94

**Table 2:** Comparison of europium and Aβ42 binding and permeation constants. Comparison of the mass of Eu^3+^ adsorption to LUVs and subsequent equilibrium dissociation constants (*K_D_*), mass adsorption of Aβ42 and apparent dye diffusion coefficient (*D*^∗^) of oligomeric Aβ42 with 0, 10 and 100 μM Eu^3+^ coordinated GM1-containing LUVs (± are the standard error of the mean).

Eu^3+^ conc. (μM)	Eu^3+^ binding (ng mm^−2^)	Eu^3+^*K_D_* (μM)	Aβ42 binding (ng mm^−2^)	*D*^∗^ (m^2^ s^−1^)
0	–	–	2.68 ± 0.54	1.72 × 10^−15^ ± 1.5 × 10^−15^
10	2.09 ± 0.69	0.48 ± 0.04	2.51 ± 0.38	2.18 × 10^−16^ ± 1.2 × 10^−16^
100	7.82 ± 0.59	0.63 ± 0.18	0.16 ± 0.11	3.92 × 10^−17^ ± 2.69 × 10^−17^

**Table 3:** Exponential function fitting for Aβ42 oligomer to the Eu^3+^-coordinated LUVs. Exponential functional expression and statistical analysis to determine mono- and bi-phasic association of the oligomeric Aβ with Eu^3+^-coordinated membranes. a0 and a1 = mono- and bi-phasic exponent per-factors respectively, the larger the per-factor signifies a larger contribution to that particular phase in the binding process. t1 and t2 = characteristic time constants for mono- and bi-phasic exponential fit (min) respectively, showing the time scale each time phase occurs. Monophasic fit columns do not have values for a1 and t2 because these are the per-factor and time constant values that only contribute to the second exponent calculated for the biphasic fits. Cor. co = correlation coefficient. RMS = root mean square.

Exponential parameter	0 μM Eu^3+^		10 μM Eu^3+^		100 μM Eu^3+^	
Mono-phasic	Bi-phasic	Mono-phasic	Bi-phasic	Mono-phasic	Bi-phasic
a0	95.11	10.7	35.1	4.4	4.9 × 10^5^	8.7 × 10^3^
a1	–	93.2	–	33.2	–	1.3 × 10^5^
t1 (min)	286.8	11	375	60	2.0 × 10^7^	9.0 × 10^5^
t2 (min)	–	430	–	505	–	9.9 × 10^5^
Chi Sq	6773.6	356.0	472.7	83.0	85.3	85.4
Cor. co	0.99	1.0	1.0	1.0	1.0	1.0
RMS% error	0.08	0.02	0.02	0.01	0.06	0.06

## Appendix A Supplementary data

Supplementary data 1 This document contains supplementary Figs. 1–3.
