# Supplementary material for: Europium as an inhibitor of Amyloid-β(1-42) induced membrane permeation
Source: FEBS Lett. 2015 Oct 24;589(21):3228–36. doi: 10.1016/j.febslet.2015.09.027 (PMC4641243; doi:10.1016/j.febslet.2015.09.027)

Supplementary information

Supplementary Figure 1. Transmission electron microscopy of Aβ42 incubated in the absence and presence of Europium. 10 μM Aβ42 was incubated with 100 μM Eu^3+^ for 48 hours and the morphology of the resulting Aβ42 species examined. A, representative image of Aβ42 in and absence of Eu^3+^, B, representative image of Aβ42 in the presence of 100 μM Eu^3+^. Scale bar = 50 μm.

Supplementary Figure 2. Mass adsorption of 0-100 μM positive control *Ctx* toxin to LUVs. 0, 0.06, 0.3, 1, 1.2, 1.5, 3, 6, and 10 μM *Ctx* was injected into the tethered LUVs. A concentration dependent increase in toxin binding was observed that reaches equilibrium at 10 μM *Ctx*. Therefore, 10 μM positive control *Ctx* was used for the experiments.

Supplementary Figure 3. Mass adsorption of 10 μM positive control *Ctx* toxin to increasing concentrations of Eu^3+^ coordinated LUVs. 0, 0.5, 5, 10, 100 and 500 μM Eu^3+^ was coordinated to tethered LUVs and 10 μM *Ctx* was administered. Between 0-10 μM Eu^3+^ showed a concentration dependent increase in ability to resist *Ctx* binding. Above 10 μM Eu^3+^ it appears that *Ctx* binding is saturated and no further inhibitory affect against the positive control is observed.

Supplementary Figure 1


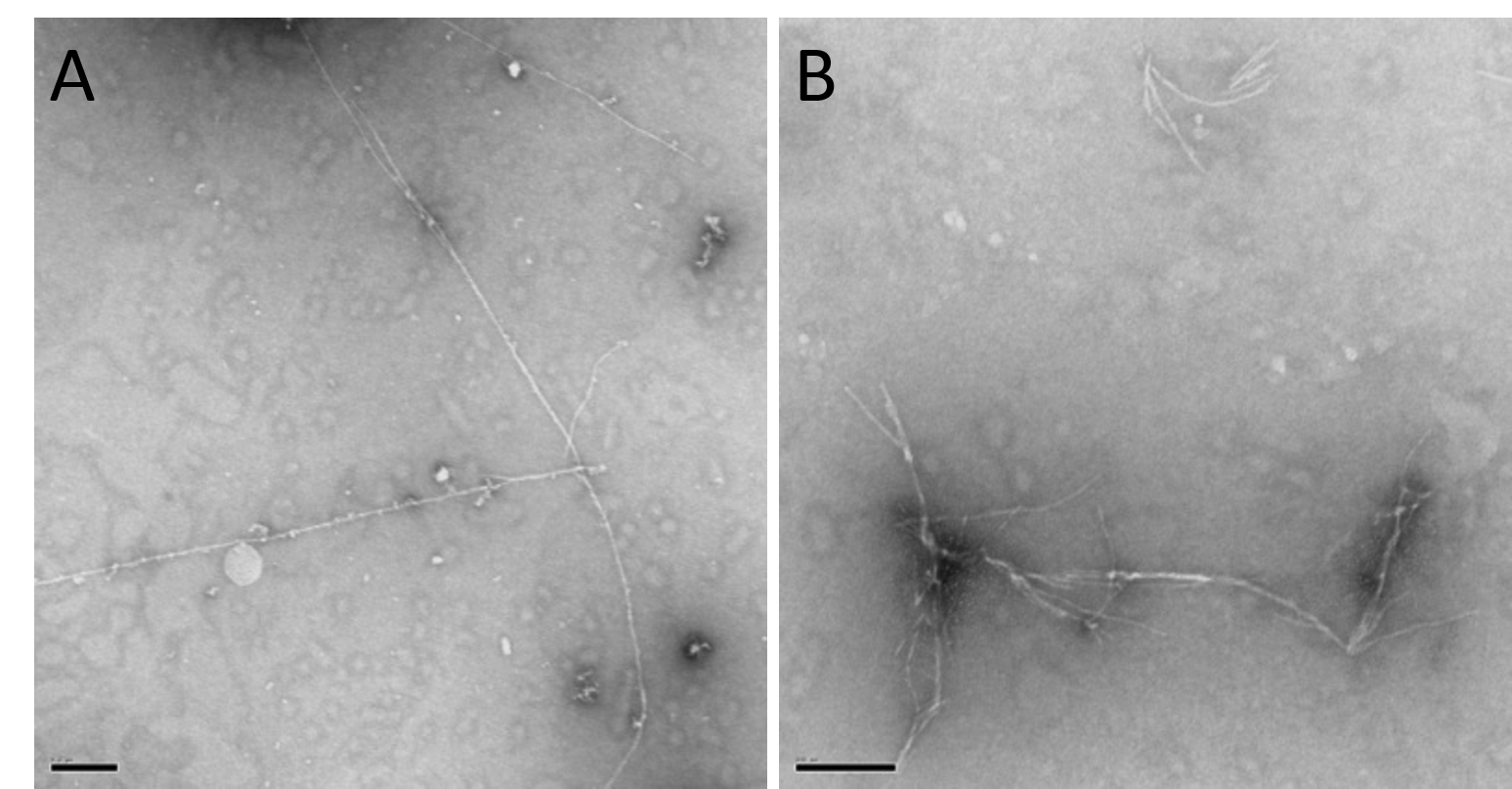


Supplementary Figure 2


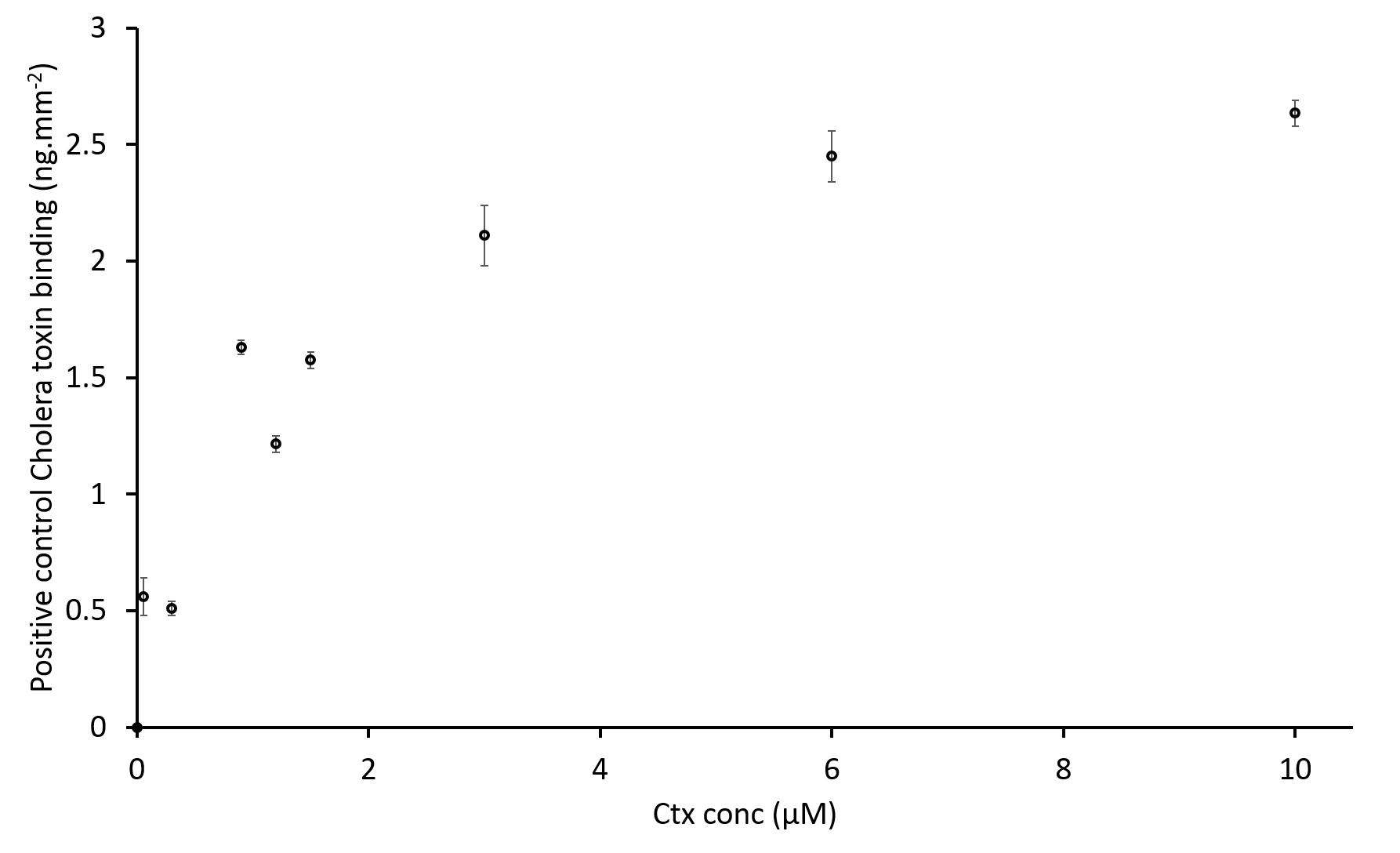


Supplementary Figure 3


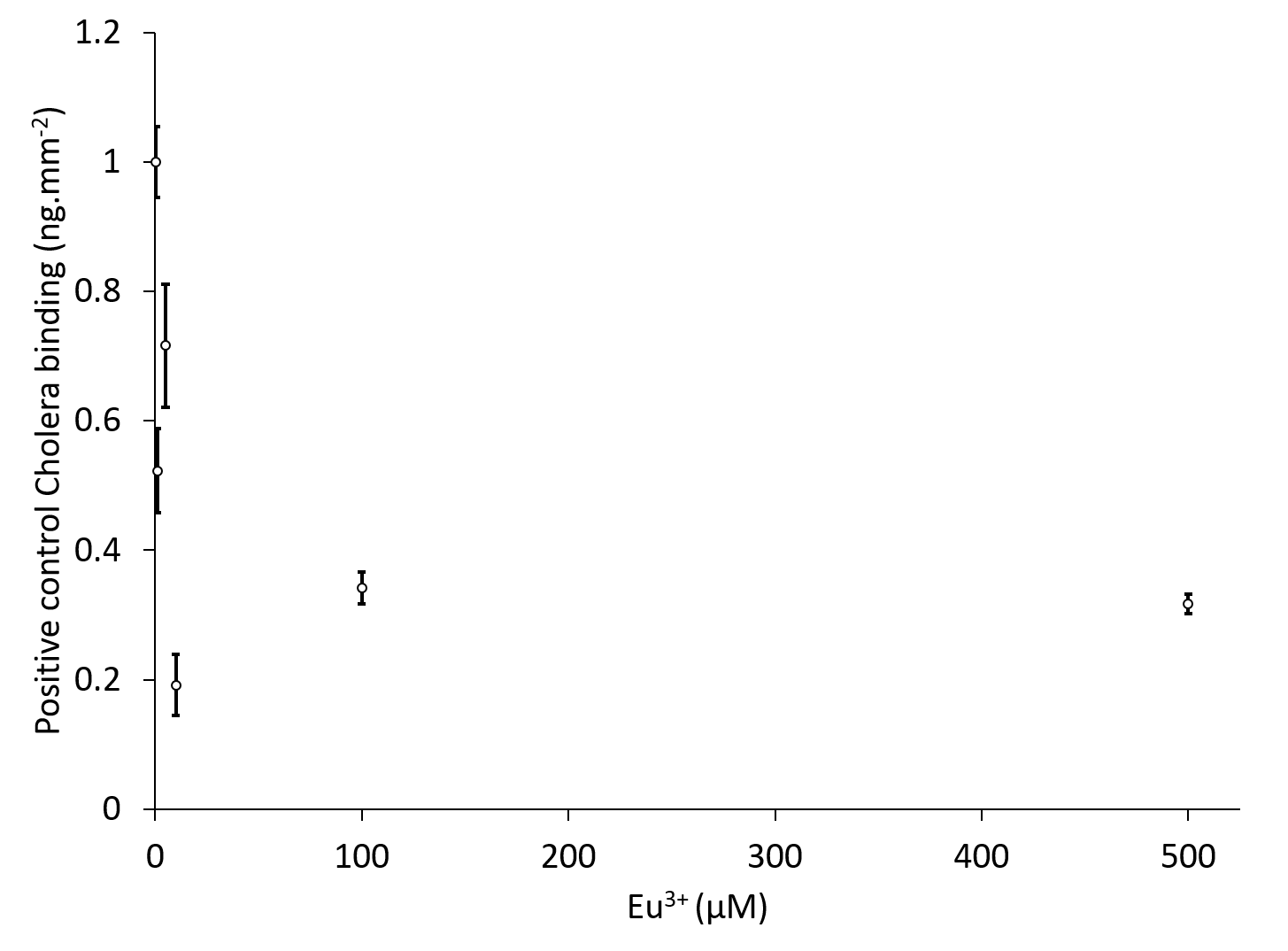

Supplement: Supplementary data 1 — This document contains supplementary Figs. 1–3. [file mmc1.docx]
